# Destruction of mesoscopic chemically modulated domains in single phase high entropy alloy via plastic deformation

**DOI:** 10.1038/s41598-022-20932-y

**Published:** 2022-10-06

**Authors:** Yoji Miyajima, Tomohiro Nagata, Kohei Takeda, Shuhei Yoshida, Satoshi Yasuno, Chihiro Watanabe, Ishikawa Kazuhiro, Hiroki Adachi, Nobuhiro Tsuji

**Affiliations:** 1 Faculty of Mechanical Engineering, Institute of Science and Engineering, Kanazawa University, Kanazawa, 920-1192 Japan; 2 Division of Mechanical Science and Engineering, Graduate School of Natural Science and Technology, Kanazawa University, Kanazawa, 920-1192 Japan; 3 Department of Materials Science and Engineering, Kyoto University, Yoshida-honmachi, Sakyo-ku, Kyoto, 606-8501 Japan; 4 Elements Strategy Initiative for Structural Materials (ESISM), Kyoto University, Yoshida-honmachi, Sakyo-ku, Kyoto, 606-8501 Japan; 5 Industrial Application and Partnership Division, Japan Synchrotron Radiation Research Institute, 1-1-1 Kouto, Sayo, Hyogo 679-5198 Japan; 6 Department of Materials and Synchrotron Radiation Engineering, Graduate School of Engineering, University of Hyogo, Himeji, 671-2280 Japan

**Keywords:** Metals and alloys, Characterization and analytical techniques

## Abstract

Chemically modulated mesoscopic domains in a fcc single phase CrMnFeCoNi equi-atomic high entropy alloy (HEA) are detected by small angle diffraction performed at a synchrotron radiation facility, whereas the mesoscopic domains cannot be detected by conventional X-ray diffraction and 2D mappings of energy dispersive X-ray spectroscopy by scanning electron microscopy and scanning transmission electron microscopy. The mesoscopic domains are deformed and shrieked, and finally destructed by plastic deformation, which is supported by the comprehensive observations/measurements, such as electrical resistivity, Vickers hardness, electron backscattering diffraction, and hard X-ray photoemission spectroscopy. The destruction of the mesoscopic domains causes the decrease in electrical resistivity via plastic deformation, so called K-effect, which is completely opposite to the normal trend of metals. We confirmed that the presence and the size of local chemical ordering or short-range order domains in the single phased HEA, and furthermore, Cr and Mn are related to form the domains.

## Introduction

High entropy alloys and medium entropy alloys (HEAs/MEAs), of which difference are depending on their mixing entropy, are widely investigated for last decades due to their excellent mechanical properties, especially at high temperatures^1,2^. Sometimes, conventional alloys bearing large fraction of alloying elements, such as Ni-based super alloys, were also treated as HEAs or MEAs One of the reasons why HEAs have widely attracted researchers is the abnormal properties caused by the multi-element, so-called “cocktail effect”^3,4^, since typical HEAs contain five or more elements. Whilst, conventional alloys consist of matrix and small fraction of alloying elements.

The physical properties of HEAs, such as electrical and magnetic properties, were also reported^5–11^, but the number of the literature is limited. The electrical resistivity, $\rho$, of HEAs at room temperature (R.T.) is range between around 0.7 m$\mu\Omega$ and around 2.2 m$\mu\Omega$, which is similar to Ni–Cr alloy^12^ known as a material for heating wires. Such high $\rho$ was attributed to the distorted lattice of HEAs that scatters free electrons^5,7^. Temperature coefficient of resistivity, the gradient of $\rho$-$T$ curves, was lower than that for the conventional alloys. Compared with the conventional alloys, the density and mobility of carrier in the HEAs were comparable and lower, respectively^6,7^. The HEAs can be either paramagnetic or ferromagnetic at room temperature, and the magnetic transformation below 100 K and the tuning of saturation magnetization were also reported^6,7,11,13^. It was also pointed out that HEAs can be used as soft magnetic materials^8^.

Short range order (SRO) domains were observed by transmission electron microscopy (TEM) in MEAs^14^. A 3D atom probe also revealed the presence of local chemical ordering (LCO) with the order of around ten atomic distance, which is longer than SRO, in MEAs^15^. The high strength of HEAs/MEAs may be achieved via LCO/SRO strengthening effects^16–18^. However, it was also reported that the effect of LCO/SRO on strength is limited/insignificant for HEAs/MEAs^19,20^. Nevertheless, it is important to assess the presence of LCO/SRO in HEAs/MEAs apart from the controversial discussion of the effect of LCO/SRO.

On the other hand, there are less numbers of reports about the plastic deformed HEAs/MEAs^21–23^. Lately, *Mazur et. al.* carefully studied the change in $\rho$ of a CrMnFeCoNi equi-atomic HEA, known as Cantor alloy^24^, upon the plastic deformation, and the SRO related phenomena “K-state” sometimes called “K-effect” was observed^9^. Unfortunately, they conducted no microstructural observations in the literature, and thus, the change in the relationship between electrical properties and microstructure on plastic deformation has not been clarified yet. In the present study, the CrMnFeCoNi equi-atomic HEA was selected as a typical fcc-HEA, and changes in $\rho$ and microstructure during cold rolling were systematically and precisely investigated. Furthermore, some techniques, such as small angle x-ray scattering (SAXS), ultra-small angle X-ray scattering (USAXS), and hard X-ray photoemission spectroscopy (HAXPES) were performed at a synchrotron radiation facility, to obtain information about domains and chemical bonding. The purpose of this study is to reveal the detail of LCO/SRO in CrMnFeCoNi equi-atomic HEA.

## Experimental results

### Microstructure of materials

**Figure 1:**
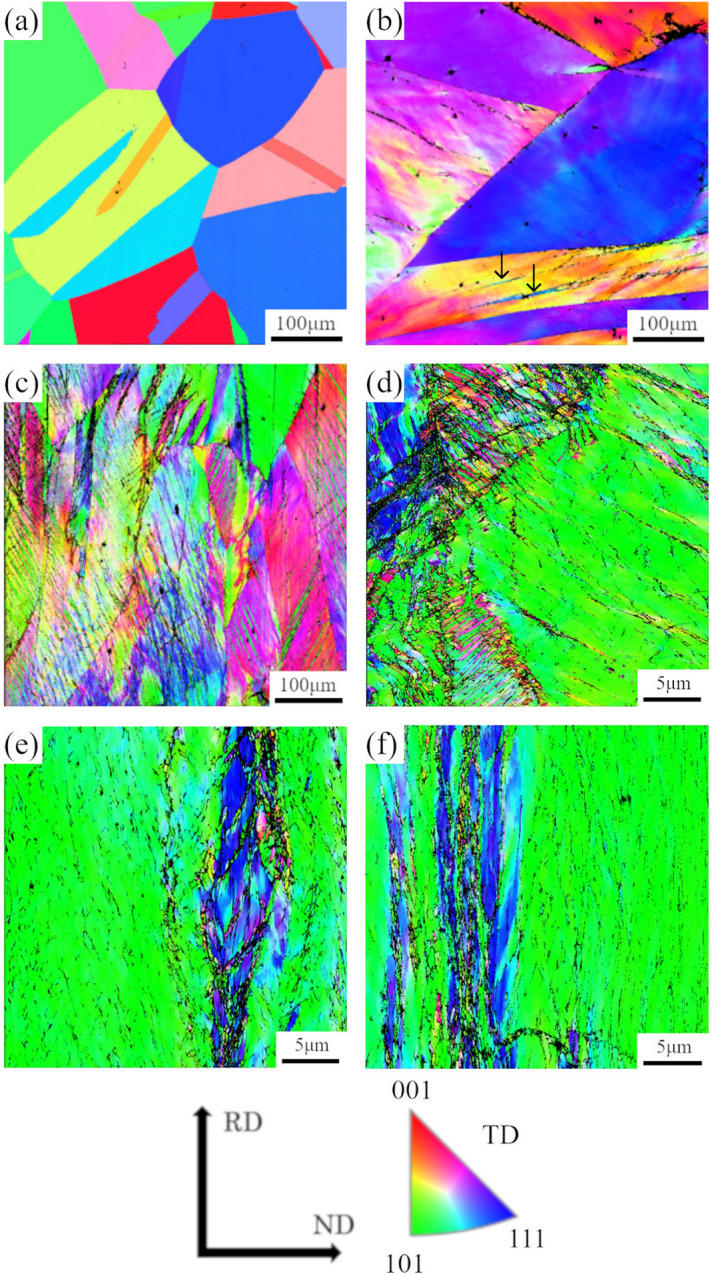
IPF maps of TD planes of CrMnFeCoNi alloy with rolling reduction of (**a**) 0%, (**b**) 20%, (**c**) 40%, (**d**) 60%, (**e**) 80%, and (**f**) 90%.

No precipitates and segregations in the rolling reception $r$ of 0% (undeformed sample) were detected by 2D mapping of the energy dispersive spectroscopy (EDS) performed with scanning electron microscopy (SEM) and scanning transmission electron microscopy (STEM). Figure 1 shows the inverse pole figure (IPF) maps of planes normal to the transverse direction (TD) of the HEA with $r$ between 0 and 90%. At $r$ = 0% (Fig. 1a), typical recrystalized microstructure having some annealing twins can be seen. The deformation of grains can be recognised since the colour of the grains has gradations at $r$ = 20% (Fig. 1b). Also, thin deformation twins shown by the black arrows can also be seen in some grains. On the further cold-rolling, grains were much deformed severely, and a high density of deformation twins can be observed at $r$ = 40% (Fig. 1c), compared with $r$ = 20%. At this stage, the initial equiaxed grains are elongated along rolling direction (RD). At $r$ = 60% (Fig. 1d), some regions contain considerably higher density of either deformation twins or shear bands, but some other regions still have a lower density of deformation twins/shear bands. It is noted that the shear bands were observed on the heavily deformed equiatomic CoCrFeMnNi HEA by cold rolling^21^. It is attributed to the difference of initial orientation of grains, even at the intermediate stage of cold-rolling. At the later stages at $r$ = 80% and 90% (Fig. 1e, f), grains were elongated clearly along RD, and some unidentified points/regions with black colour aligned along RD, that is thought to be the deformation twins/shear bands. The unidentified points/regions are attributed to the smaller twin width or grain size than the step size of EBSD, since such situation results in the failure of the determination of orientation by EBSD.

**Figure 2:**
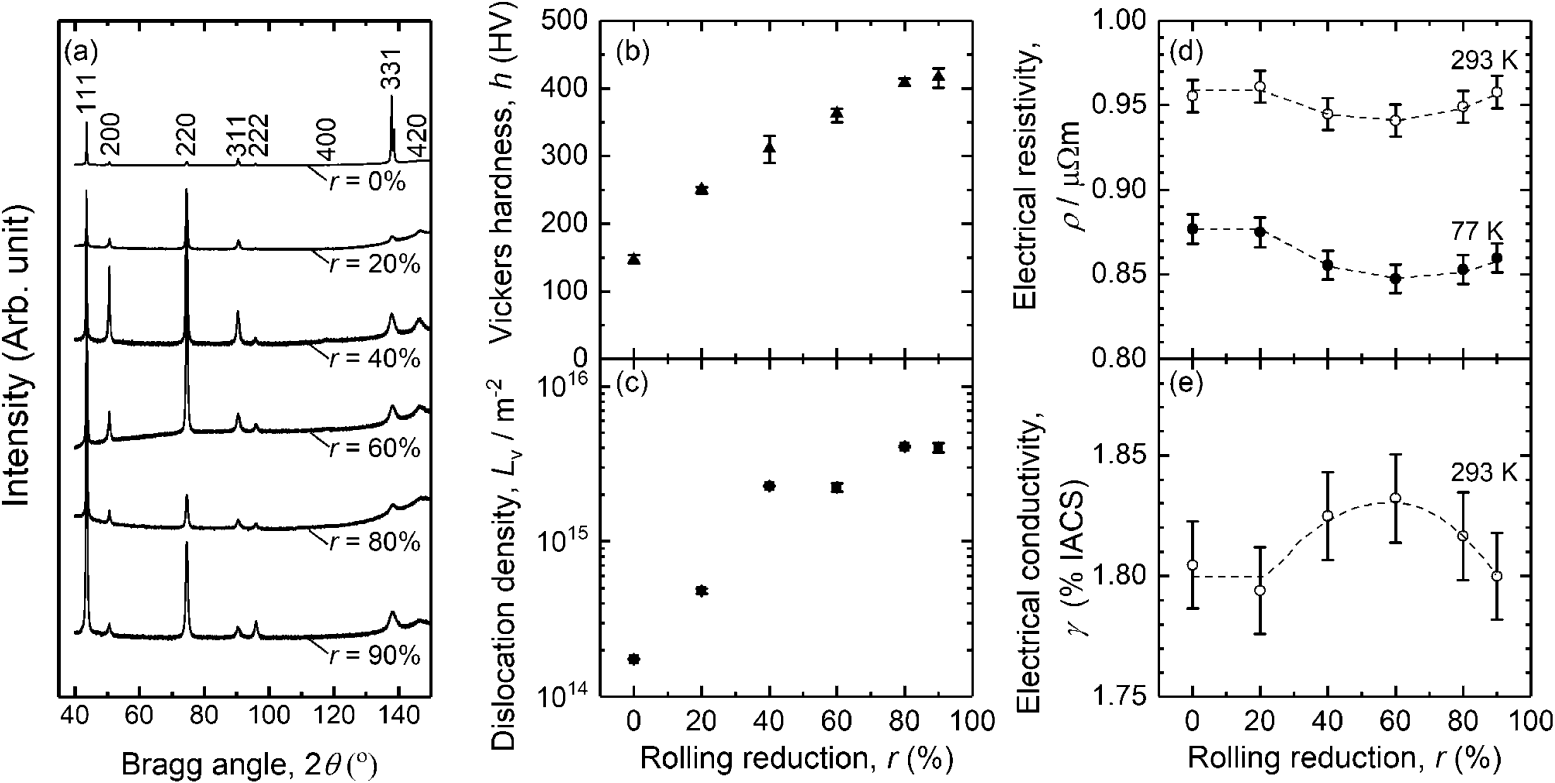
Rolling reduction dependence with the rolling reduction between 0 and 90% of CrMnFeCoNi alloy, (**a**) XRD spectra, (**b**) Vickers hardness, (**c**) dislocation density evaluated using XRD data, (**d**) electrical resistivity at 77 K and 293 K, and (**e**) electrical conductivity at 293 K.

Figure 2a shows X-ray diffraction (XRD) profile of the HEA depending on $r$. The HEA is fcc single phase for any $r$, and the broadening of the peaks occurs with increasing $r$. The broadening of XRD peaks upon plastic deformation often means the increase in dislocation density ${L}_{\mathrm{V}}$. Figure 2b shows the change in Vickers hardness of the CrMnFeCoNi HEA on the cold rolling. Vickers hardness increases about 270 HV from 146 HV at $r$ = 0% to 417 HV at $r$ = 90%, which can be attributed to the grain subdivision shown in Fig. 1.

Figure 2c shows $r$ dependence on ${L}_{\mathrm{V}}$ of the HEA. ${L}_{\mathrm{V}}$ was determined using the modified method (combination of modified WH and modified WA) and the reported elastic constants of a CrMnFeCoNi alloy^25^. The error bars represent the standard error of the linear fittings, but the deviation is almost within the size of the symbols. The value of ${L}_{\mathrm{V}}$ ~ 2 × 10^14^ m^−2^ at $r$ = 0% should be treated as a reference since the grain size may be too large for XRD profile analysis. ${L}_{\mathrm{V}}$ gradually increases from about $2\times {10}^{14}$ m^−2^ at $r$ = 0% to about $5\times {10}^{14}$ m^−2^ at $r$ = 20%. Then, ${L}_{\mathrm{V}}$ increases to around $2\times {10}^{15}$ at $r$ = 40% and 60%. ${L}_{\mathrm{V}}$ finally increases to be around $4\times {10}^{15}$ and stays almost constant after $r$ = 80%.

### Electrical resistivity measurement

Dependencies of the specific resistivity measured at 293 K (R.T.) ${\rho }_{293}$ and 77 K ${\rho }_{77}$ on $r$ are shown in Fig. 2d. The ${\rho }_{293}$ and ${\rho }_{77}$ are around 0.95 m$\mu\Omega$ and around 0.86 m$\mu\Omega$, respectively. ${\rho }_{293}$ is always about 0.09 m$\mu\Omega$ higher than ${\rho }_{77}$ due to the contribution of the lattice vibration . Initially, ${\rho }_{77}$ is around 0.88 m$\mu\Omega$, but it is almost unchanged to be around 0.88 m$\mu\Omega$ at $r$ = 20%. Then, ${\rho }_{77}$ decreases down to around 0.85 m$\mu\Omega$ at $r$ = 60%. When $r$ exceeded 60%, ${\rho }_{77}$ increases up to 0.86 m$\mu\Omega$ until $r$ of 90%.

From electrical materials point of view, electrical conductivity at 293 K, $\gamma$ %IACS, is important, and thus shown in Fig. 2e. It is noted that $\gamma$ is converted from ${\rho }_{293}$. $\gamma$ is initially at around 1.80%IACS at $r$ up to 20%, and then, increases to around 1.83%IACS at $r$ = 60%. Finally, $\gamma$ decreases down to around 1.80%IACS at $r$ = 90%.

### Small angle diffraction

**Figure 3:**
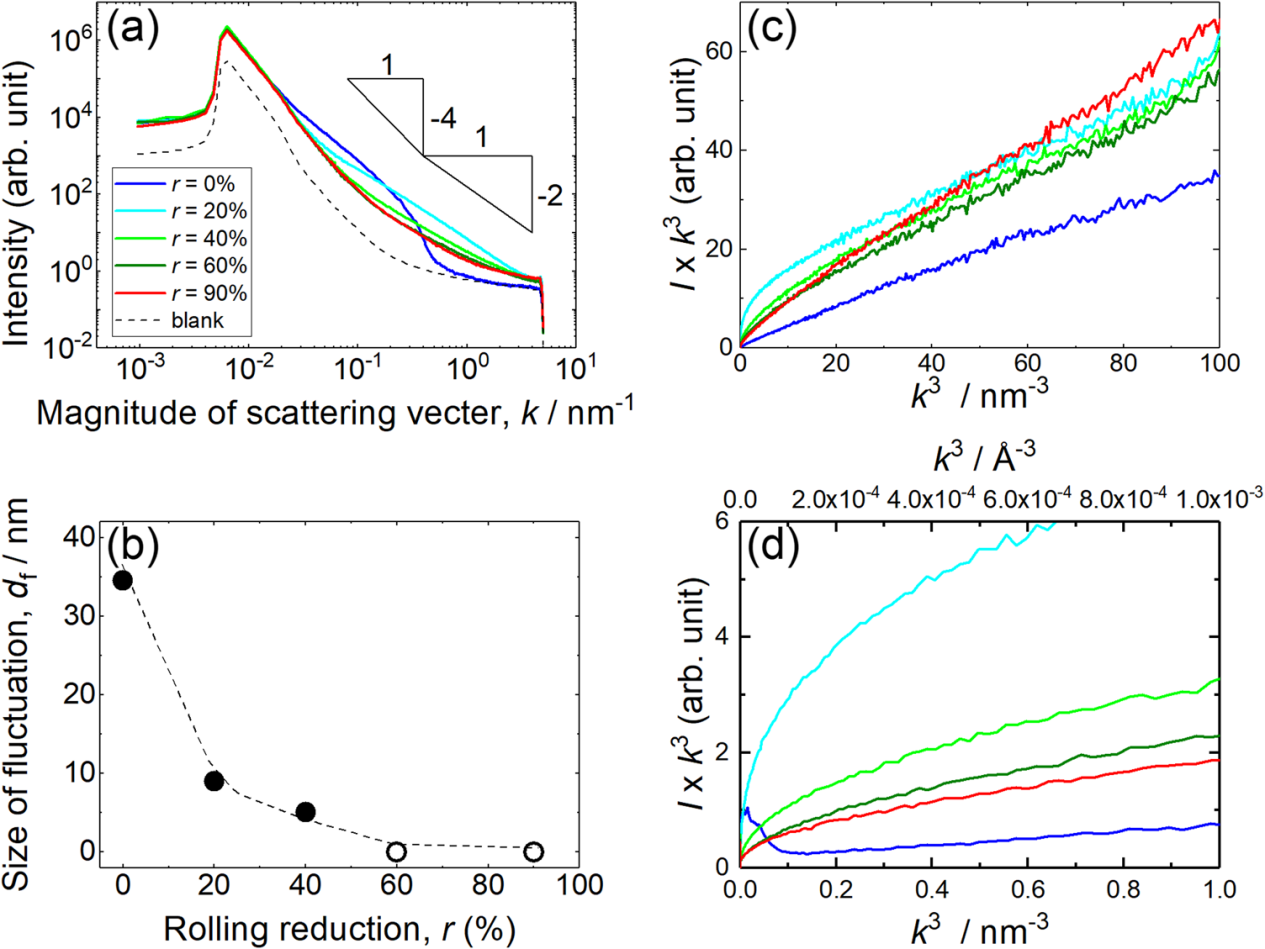
Rolling reduction dependence of (**a**) scattering intensity versus magnitude of scattering vector for SAXS and (**b**) size of fluctuation evaluated from SAXS. The triangles in (**a**) represent the gradient of ${k}^{-4}$ and ${k}^{-2}$. It is noted that white filled circles in (**b**) represents that the size of fluctuation is too small to be measured by SAXS. It is noted that the blank curve is shown as a reference. (**c**) $I \times {k}^{3}$ versus ${k}^{3}$ plot of CrMnFeCoNi equi-atomic HEA and (**d**) magnified plot close to the original point of (**a**).

Figure 3a is the scattering vector, $k=\left(4\pi \mathrm{sin}\theta \right)/\lambda$, dependence of scattering intensity obtained by both SAXS and USAXS. Here, $\theta$ and $\lambda$ are the scattering angle and wavelength of the incident X-ray, respectively. The dashed line is for the blank shown as just a reference and should not be compared with the intensity of the measured data.

Lower and higher $k$ regions contain the information of size and shape of fluctuation region of electron density, respectively. In the case of $r$ = 0%, increase in scattering intensity was observed with $k$ between 0.02 nm^−1^ and 0.6 nm^−1^ compared with the blank. For $r$ of 20% and 40%, less increase in the $k$ region can be recognized compared with $r$ of 0%. The gradient in the $k$ region with $r$ of 60% and 90%, are similar to that for blank, thus it can be considered that the origin of the scattering is almost disappeared at around $r$ of 60%. Strictly speaking, slight increase at $k$ of around 1 nm^−1^ can be seen.

As shown in Fig. 3b, guinier diameter: ${d}_{f}$ can be evaluated from Guiner plot, which is scattering intensity in natural log versus ${k}^{2}$. When the peak appears in higher $k$, size of fluctuation region becomes smaller, and vice versa. The size of fluctuation area becomes smaller with increasing $r$, since the centre position of the increase in scattering intensity shifts toward higher $k$. The initial size of ${d}_{f}$ is about 35 nm at $r$ = 0%. ${d}_{f}$ decreases down to 9 nm and 5 nm for $r$ = 20% and 40%, respectively. ${d}_{f}$ at $r$ of 60% and 90% cannot be obtained since the size is too small, thus, ${d}_{f}$ is treated as zero.

As shown in Fig. 3c, SAXS/USAXS data can be plotted like $I \times {k}^{3}$ versus ${k}^{3}$ following Porod’s law^26,27^. This plot can give the information about the interface between the fluctuated region and matrix. If the interface is clear like precipitates in conventional alloys, $I \times {k}^{3}$ should initially increase with increasing ${k}^{3}$, and then, reaches to a constant value region at higher ${k}^{3}$. However, such constant region cannot be recognized on Fig. 3c, d It is noted that Fig. 3d is displayed to clarify that the constant region cannot be seen close to the original points. Consequently, the boundary between the fluctuated region and the matrix is thought to be diffusive rather than discontinuous like precipitates in conventional alloys.

### Photoemission spectroscopy

**Figure 4:**
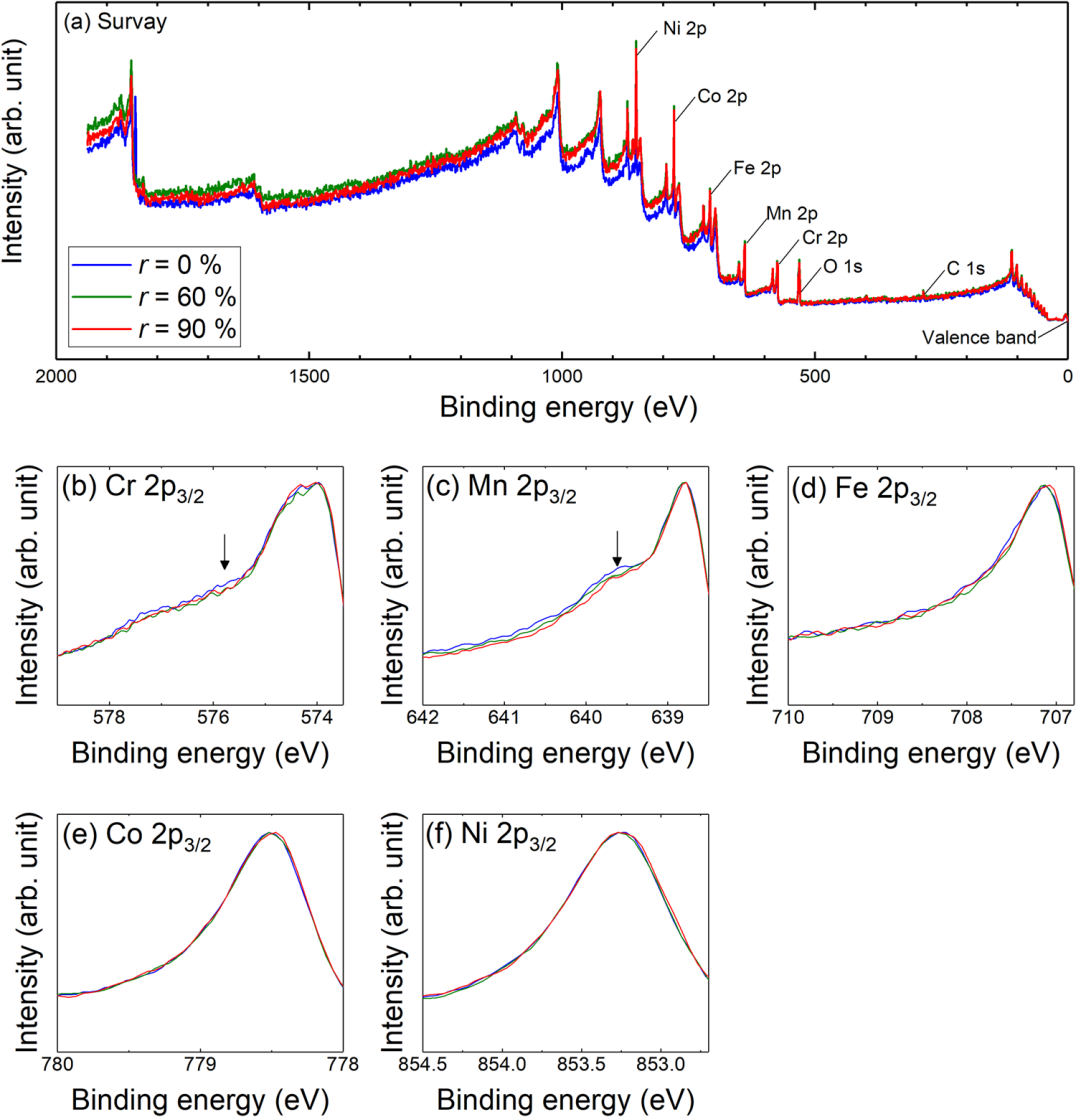
HAXPES spectra of (**a**) wide range survey with the bonding energy up to about 2000 eV, (**b**) Cr 2p_3/2_, (**c**) Mn 2p_3/2_, (**d**) Fe 2p_3/2_, (**e**) Co 2p_3/2_, and (**f**) Ni 2p_3/2_. The arrows in (**b**) and (**d**) are the guide of the eyes.

Figure 4a shows the wide range survey spectra of HAXPES, and the peaks of Ni, Co, Fe, Mn and Cr can be recognized in addition to the C 1 s and O 1 s peaks. The expanded data at around 2p_3/2_ for Ni, Co, Fe, Mn and Cr are shown in Fig. 4b. The peak of Cr and Mn seems to have slight $r$ dependence at the shoulder of the higher bonding energy side of 2p_3/2_ (left hand side of the peaks in Fig. 4b). In other words, both peaks have higher intensity at the left-hand side of the peak when $r$ = 0%. The high intensity shoulder seems to be reduced with increasing $r$. Whereas, the 2p_3/2_ peak for Fe, Co, and Ni seems almost identical for different $r$. It is clearly said that the difference in 2p_3/2_ peak of Cr and Mn are not associated with the oxide, since the effect of oxide should appear much higher bonding energy regions. Thus, HAXPES result indicates that Cr and Mn shows different behavior due to the cold rolling, whereas Fe, Co and Ni seem independent from the cold rolling.

## Discussion

From Fig. 1, the grain refinement progresses with increasing $r$, which is attributed to grain subdivision^28^. Grain refinement in the present study can consist of three types: formation of subgrains, subdivision due to deformation twins, and formation of ultra-fine grains within shear bands. Such microstructure evolution observed in this study agrees with the detailed microstructure and texture analysis by SEM/EBSD on cold rolled CoCrFeMnNi HEA with the rolling reduction of 90%^21^. The shear band is also confirmed in the report. The formation of deformation twins is attributed to the low SFE of HEA, and the texture analysis, such as the presence of the Brass orientation. Nevertheless, it can be clearly said that the density of GB which can be the scattering centres of free electrons increases with increasing $r$.

In the case of MEAs/HEAs, the WH and modified method (including convolutional multiple whole profile) has been already applied by some researchers^29,30^. Thus, it was assumed that determined ${L}_{\mathrm{V}}$ via XRD peak analysis by the modified method is still applicable for Cantor alloy, at least, change in ${L}_{\mathrm{V}}$ (${\Delta L}_{\mathrm{V}}$) compared with $r$ = 0% is meaningful. Initial stage of rolling, ${L}_{\mathrm{V}}$ drastically increases to ~ 2 × 10^15^ m^−2^ at $r$ = 40%. Then, ${L}_{\mathrm{V}}$ gradually increases to be ~ 4 × 10^15^ m^−2^ with increasing $r$ up to 90%. Nevertheless, ${L}_{\mathrm{V}}$ is considered to increas with increasing $r$ as shown in Fig. 2c. Therefore, the trend of the Vickers hardness, as shown in Fig. 1, agrees with the microstructure evolution based on the EBSD and XRD measurements.

As shown in Fig. 2d, the values of ${\rho }_{293}$ and resulting values of $\gamma$ is similar to the reported value of HEAs^5,7–10^ and conventional alloys Ni–Cr alloy^12^ and Ni base super alloy (Alloy625)^31^. It is noted that Ni base super alloy is sometimes classified to MEA. In the case of such Ni alloys, matrix is classified to disordered phase, thus, the distorted lattice in HEAs/MEAs can be similar situation of conventional disordered alloys having relatively high $\rho$.

In general, the $\rho$ of pure metals and conventional alloys increases as plastic deformation progresses, such as pure Cu^32^, pure Ni^33^, pure Al^34^, and Al alloys^34,35^. These electrical responses are strongly associated with the increase in ${L}_{\mathrm{V}}$ and density of grain boundary ${S}_{\mathrm{V}}$, since dislocations and grain boundaries work as scattering centres of free electrons in alloys.

However, the changes in $\rho$ of the HEA with $r$ up to 60% are different from the conventional pure metals and alloys. This discrepancy in electrical behavior agrees well with the literature by *Mazur *et al.^9^. The reduction in $\rho$ of a CrMnFeCoNi equi-atomic HEA due to rolling is attributed to the K-state which is sometimes called K-effect instead. Such reduction of $\rho$ due to the K-effect is ascribed to the destruction of SRO by the plastic deformation, which is also observed not only in conventional Ni alloys^36^ but also in Alloy 625^31^.

In the case of conventional alloy, the reduction of $\rho$ is attributed to the destruction of SRO micro domains which are proposed based on the X-ray analysis^37^. TEM observation and Monte Carlo simulation also support the presence of the SRO domains in binary alloys showing order–disorder transition^38,39^. Anyhow, electrical resistivity measurement of HEA indicates the presence of SRO domains in microstructure. The cause of characteristic electrical behaviour of the HEA will be discussed by assuming that the HEA has LCO/SRO domains, since the existence of LCO/SRO was experimentally confirmed in MEAs^14,15^.

**Figure 5:**
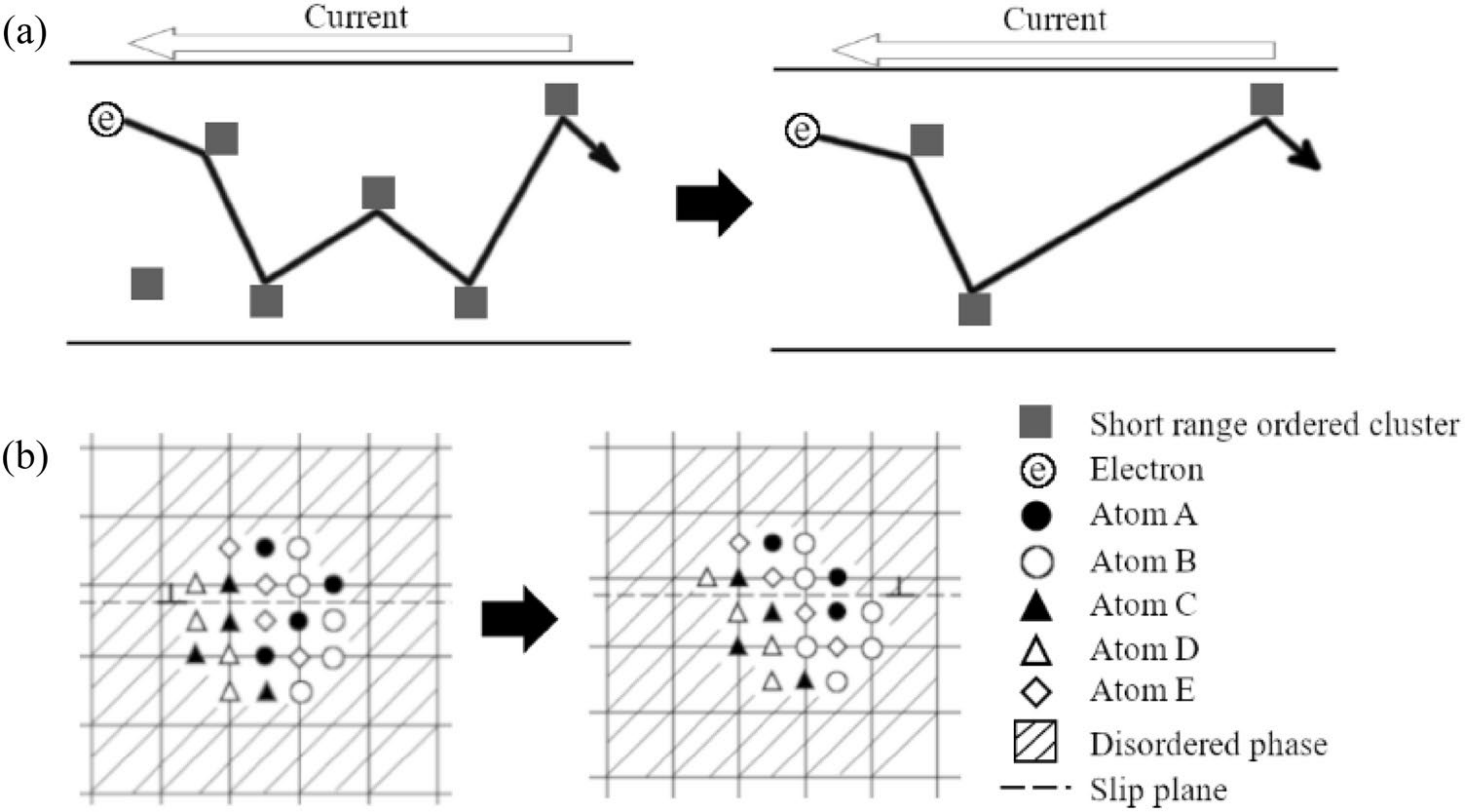
Schematic diagram of a binary alloy. (**a**) scattering of free electrons by short range ordered domains and (**b**) cut of short range ordered domain by dislocation due to plastic deformation. It is the modification from the binary alloy case ^31^.

Increase in $\rho$ is often explained by the increase of the concentration of the alloying elements in matrix due to the dissolve of precipitates via cutting by dislocations. However, it is not valid in the case of K-effect in HEAs/MEAs, since the matrix of HEAs/MEAs is almost completely mixed. Thus, cutting of LCO/SRO results in local atomic movement within several atomic distance as shown in Fig. 5. The destruction of LCO/SRO should regard as rather locally occurring order–disorder transition via plastic deformation.

Once the destruction of LCO/SRP is completed, decrease in ${\rho }_{77}$ cannot occur anymore. ${\rho }_{77}$ showed characteristic behaviour of initial decreasing and then increasing before and after $r$ = 60%, as shown in Fig. 2d. The increase in ${\rho }_{77}$ can be connected with the increase in the lattice defect density (${L}_{\mathrm{V}}$ and ${S}_{\mathrm{V}}$). The increase in lattice defect density clearly occurs based on the microstructure observations by FE-SEM/EBSD (Fig. 1) and analysis of XRD peak broadening (Fig. 2c).

The large ${d}_{f}$ detected by SAXS/USAXS can be associated with the presence of slight modulation of the concentration of the elements with the order of a few tens nm. Actually, 3D atom probe measurement revealed the existence of Cr rich and (Co + Ni) rich layers in CoCrNi MEA^15^. Thus, similar modulation may exist in the in CrMnFeCoNi equi-atomic HEA. However, even if there is such slight modulation on the concentration of the elements, the magnitude of the modulation on the concentration is quite small. Since the fluctuation regions with ${d}_{f}$ of 35 nm at $r$ = 0% should be large enough to be recognized with this magnification using STEM/EDS 2D-mapping.

As contrary, it is possible to obtain the information about the shape of fluctuation regions with higher region of $k$ above around 0.1 nm^−1^ in Fig. 3a. The scattering intensity changes with ${k}^{-4}$ and ${k}^{-2}$ for spherical and disk shapes, respectively. Thus, the shape at $r$ = 0% is spherical, whereas the shape at $r$ = 20% and 40% is elongated like a disk. As a conclusion, the fluctuation region elongated from the spherical shape with increasing $r$, and ${d}_{f}$ decreases from about 35 nm down to 0 nm at $r$ = 60%.

However, the initial size of ${d}_{f}$ (Fig. 3b) is debatable. For instance, the size of LCO in MEA is around 2 nm from the 3D atom probe measurement^15^. The average size of SRO domains appearing in MEA was around 1 nm based on the TEM observation^14^. Since the specimen used in the present study is a HEA, there can be such mesoscopic order LCO domains. Such size of region cannot be said SRO anymore. From Fig. 3c, d, the fluctuation area found by SAXS/USAXS may have diffusive boundary which is different from clear/sharp boundary like precipitates/matrix in the conventional alloys. It is noted that the constant region starts is depending on the material.

HAXPES gives information about the chemical bonding of the specimen. For instance, when there is a specific chemical bonding, such as metal–oxygen bonding, a new peak can be seen at a specific bonding energy. However, such strong peak cannot be seen for metal bonding compared with the covalent bonding like metal–oxygen bonding. However, even if the metal bonding gives less intensity on HAXPES, there should be a difference on a peak when the metallic bonding configuration is changed by some reasons.

For instance, the high intensity shoulder was reported on Mn 2p_3/2_ peak of Al-Mn alloys. The shoulder is about 1 eV higher than Mn 2p_3/2_ peak, which is associated with the change of chemical composition of Al-Mn alloys. In other words, the high intensity shoulder is related to the chemical bonding. Thus, it is thought that the high intensity shoulder appears in Cr and Mn peaks measured by HAXPES are related to the chemical bonding^40^.

From HAXPES (Fig. 4), any changes related to the chemical bonding must appear as the change of the shape of Cr and Mn peaks. Of course, the chemical composition cannot be changed due to the plastic deformation. However, the local configuration of atoms can be modulated due to the plastic deformation via dislocation activities. Such change of local configuration of atoms (metallic bonding) gives the change of the Cr and Mn peaks in HAXPES. Thus, local configuration of Cr and Mn around LCO/SRO domains are changed (see Fig. 5), which agrees with the occurrence of K-effect.

Cr and Mn rich/poor region can exist in CrMnFeCoNi equi-atomic HEA based on the HAXPES results, and the fluctuation regions are distinguished by the density of electrons from SAXS/USAXS measurement point of view. In the CrMnFeCoNi alloy case, composed elements and their number of electrons lined up in the periodical table as the element is written. Thus, the fluctuation regions are related by Cr and Mn. The 3D atom probe measurement on the CoCrNi MEA indicates that Cr rich region appears^15^, and therefore the presence of Cr and Mn rich region in CrMnFeCoNi equi-atomic HEA cannot be immediately denied.

Even though the mesoscopic-order domains exist, the change in concentration of Cr and Mn must be quite small since STEM/EDS 2D-mapping show almost homogeneous distribution of all elements. The result of HAXPES may imply that there are specific chemical bonding related to Cr and Mn, which enhance either forming LCO/SRO or modulation of concentration for Cr an Mn.

## Conclusions

Comprehensive observations/measurements were adopted for a CrMnFeCoNi equi-atomic HEA in order to connect the change in electrical resistivity and microstructure caused by plastic deformation. SAXS/USAXS implies that there are chemically modulated mesoscopic domains. The fluctuation regions can be associated with fluctuation of the concentration of certain elements, though the modulation of the concentration of the constituent elements cannot be detected by STEM/EDS 2D mappings. Furthermore, both regions can be also destructed and elongated by the plastic deformation like cold rolling. HAXPES measurement suggests that Cr and Mn have specific chemical bonding which can be destructed by plastic deformation, which may relate either LCO/SRO or fluctuation area, or both. The decrease in electrical resistivity with increasing $r$, so called K-effect, is associated with the reduction of the chemically modulated mesoscopic domains.

## Methods

### Sample preparation

In the present study, a CrMnFeCoNi equi-atomic HEA was fabricated by vacuum induction melting. The chemical composition of the alloy is shown in Table 1. The size of the ingot was about $\phi$ = 78 mm $\times$ 140 mm, and then, hot-forged at 1423 K, resulting in a plate shape with 15 mm in thickness, 150 mm in width, and 200 mm in length. The scale at surfaces and feeding head were removed, and then, 3 mm thick plates with the width of about 10 mm and length of about 30 mm were cut by an arc discharge cutting machine. The plates were vacuum-sealed in quartz tubes and solution-treated at 1473 K for 10.8 ks using a tube furnace FTSS-520 (Tokyo Garasu Kikai) and then quenched into cold water.

**Table 1:** Composition of CrMnFeCoNi alloy.

Composition	Cr	Mn	Fe	Co	Ni	C	S	O	N
(mass%)	18.42	19.39	19.86	20.76	20.86	0.007	0.0062	0.0100	0.0014
(at.%)	20.01	19.94	20.09	19.90	20.07	–	–	–	–

The solution-treated plates were cold-rolled using a rolling mill, with the roll having a diameter of 100 mm (Nihon cross atsuen) without lubrication. The rolling reduction $r$ (%) is defined as thickness reduction divided by the initial thickness. The rolling was performed up to $r$ = 90%. Hereafter, the specimen without rolling is referred to as $r$ = 0%. The sample coordinates were defined as rolling direction (RD), transverse direction (TD), and normal direction (ND). Planes normal to RD, TD, and ND were denoted as RD, TD, and ND planes, respectively.

### Vickers hardness tests

The Vickers hardness was measured using an HMV-G30 (SIMADZU) on ND planes of the pre- and post-rolled specimens. The load and duration of the hardness measurement were set to be 5 kgf and 10 s, respectively. Seven points were totally measured, and both maximum and minimum values were excluded, and the remaining five values were used to obtain their average.

### Microstructure observations by TEM and SEM/EBSD

The specimens for microstructural observations with a field emission-scanning electron microscope/electron backscattered diffraction (FE-SEM/EBSD) were cut from the rolled sheets using the arc discharge cutting machine. The damaged layer of the surface was removed by emery paper up to #2000, and then electrolytic polishing in the nital solution (nitric acid and methanol mixture with 1:2 in volume) with a voltage of 6 V at 233 K for about 120 s. FE-SEM/EBSD observations were performed using JSM-7900F (JEOL) with the "symmetry" detector controlled by Aztec software (Oxford instruments). The EBSD measurements were performed with the acceleration voltage of 20 kV. The step size of EBSD was set to be 0.5 $\mu$m for $r$ is lower than 40%, and $\le$ 50 nm for $r$ is higher than 40%.

### EDS observations

Energy-dispersive X-ray spectroscopy (EDS) was performed with SEM, and a JSM-6390A (JEOL) equipped with a JED-2300 EDS detector (JEOL) was used. EDS was also performed with scanning transmission electron microscopy (STEM), and a JEM-2100F (JEOL) equipped with a JED-2300 T EDS detector (JEOL) was used. The EDS observations were performed to check the presence of segregation in as-quenched specimen before cold rolling ($r$ = 0%). The acceleration voltage used was 20 kV for SEM and 200 kV for STEM. The Cr K, Fe K, Mn K and Ni K lines were used for the EDS 2D mapping. The specimen for SEM/EDS was fabricated by the same procedure for EBSD measurements. The foil specimens for the STEM/EDS observations were prepared by mechanical polishing using emery paper. They are finished by electrolytic polishing using a Tenupole-5 (Struers) twin-jet polisher with a mixture of perchloric acid, glycerine, and methanol (10%, 20%, and 70%, in volume) at 243 K, with the voltage of 15 V.

### XRD measurements

X-ray diffraction (XRD) measurements were performed using X’Pert PRO MPD (PANalytical) with Cu tube (*λ* = 0.15418 nm) to determine phase and dislocation density ${L}_{\mathrm{V}}$. The voltage and the current of the Cu tube were 45 kV and 40 mA, respectively. The ND plane was selected for the XRD measurements, and a side of the sheet was mechanically polished by emery papers until the thickness was reduced to be 75%. Four peaks (111, 200, 220 and 311) are used for the analysis.

The change in ${L}_{\mathrm{V}}$ can be determined by some methods, such as Williamson-Hall (WH) method^41,42^, Warren-Averbuch (WA) method^43,44^, and their modified method (modified WH, modified WA, and multiple whole profile fitting)^45–47^. WH method uses the WH plot using following relationship.

1 $$\frac{\left(\Delta 2\theta \right)\mathrm{cos}{\theta }_{\mathrm{B}}}{\lambda }=\frac{{K}_{\mathrm{s}}}{D}+\frac{2e\mathrm{sin}{\theta }_{\mathrm{B}}}{\lambda }$$

Here, $\Delta 2\theta$ is the full width-half maximum, ${\theta }_{\mathrm{B}}$ is the position of Bragg peak, $e$ is the microstrain, ${K}_{\mathrm{s}}$ is the Scherrer constant which is normally 0.9, and $D$ is the crystallite size. After obtaining $e$ of materials from the slope of the WH plot, $e$ is converted to ${L}_{\mathrm{V}}$ using the following relationship^48^.

2 $${L}_{\mathrm{v}}=\frac{K{e}^{2}}{{b}^{2}}$$

Here, $\mathrm{K}$ is a numerical constant depending on the crystal structure (16.1 for fcc), and $b$ is the magnitude of Burgers vector. The WA method is based on the following equation.

3 $$\mathrm{ln}A\left(L\right)=\mathrm{ln}{A}^{s}\left(L\right)+\mathrm{ln}{A}^{D}\left(L\right)$$

Here, $A\left(L\right)$ is the real part of the Fourier coefficient of the Bragg peak. ${A}^{s}\left(L\right)$ and ${A}^{D}\left(L\right)$ are the size and distortion contributions, respectively. $L$ is the Fourier length which appears from the Fourier transformation. From ${A}^{D}\left(L\right)$, ${L}_{\mathrm{V}}$ can be obtained.

In the case of modified method, contrast factor $C$ which is the correction term of the dislocation system is introduced^45–47^. Thus, modified WH plot and modified WA plot gives better fitting compared with the original WH and WA plots, since the elastic anisotropy of dislocations is taken into account. In the case of modified WH plot, following equation is used with the average contrast factor $\overline{C }$.

4 $$\Delta K=\frac{0.9}{D}+\alpha {\left({L}_{\mathrm{V}}\right)}^{1/2}\left(K\cdot {\overline{C} }^{1/2}\right)+O\left\{{K}^{2}C\right\}$$

Here, $\alpha$ is a constant depending on the effective outer cut-of radius of dislocations, $O$ is the higher order term. ${K}_{\mathrm{s}}=0.9$, $\Delta K=\left\{\left(\Delta 2\theta \right)\mathrm{cos}{\theta }_{\mathrm{B}}\right\}/\lambda$ and $K=\left(2\mathrm{sin}{\theta }_{\mathrm{B}}\right)/\lambda$ are used.

In the case of modified WA method, Eq. (3) is modified to be Eq. (5).

5 $${\text{ln}}A\left( L \right) \cong {\text{ln}}A^{s} \left( L \right) - \left( {L_{{\text{V}}} } \right)\frac{{\pi b^{2} }}{2}L^{2} {\text{ln}}\left( {\frac{{R_{{\text{e}}} }}{L}} \right)\{ K^{2} \bar{C}\} + O\left( {K^{4} C^{2} } \right)$$

Here, ${R}_{\mathrm{e}}$ is the effective cut-of radius of dislocations, and $O$ is the higher order term.

It should also be noted that the modified method introduces new parameters on the arrangement of dislocations $M={R}_{\mathrm{e}}{\left({L}_{\mathrm{V}}\right)}^{1/2}$^46^, in addition to ${L}_{\mathrm{V}}$. Thus, the constant value of ${L}_{\mathrm{V}}$ may be concerned with the change in the arrangement of dislocations.

### Electrical resistivity measurements

Electrical resistivity measurements were performed using a four-terminal method to determine $\rho$ precisely. The two terminals were for current, and the other two terminals located between the two current terminals were for measuring voltage drop. Since the internal resistance of a nano-voltmeter located between the internal two wires is much higher than the metal specimen, e.g. the order of M $\Omega$ for the internal resistance, almost all current passing through the specimen instead of the voltmeter, and the contact resistance between wires and the specimen and resistance of the wires between a voltmeter can be ignored. Four pure Ni wires with the diameter of 0.3 mm were spot welded using a spot welder NRW-100 W (Nippon Avionics) and a welding head NA-60A (Nippon Avionics). In order to avoid the damage and delamination at the spot-welded contacts, 3D printed ABS resin frame was used. The Ni wires were wrapped with the frame, and therefore the spot-welded contacts can avoid receiving the critical force which occurs during handlings, preparations and electrical measurements. The detail of the shape of the flame can be found elsewhere^49^. Using a precise current source 6220 (Keithley) and a nanovoltmeter 2182A (Keithley), electrical measurements were conducted at 77 K (in liquid nitrogen) and at 293 K (ambient condition) with a constant current of 100 mA. The passing current direction was automatically and alternatively changed during the measurements in order to remove the effect of the thermo-electromotive force appearing at dissimilar metal contacts in the circuit. Finally, the obtained values of $\rho$ was converted from electrical resistance $R$, using the cross-sectional area of the bar-shaped specimen and the distance between the internal two Ni wires. The errors caused by the measurements of the specimen were less than 1%, and represented as error bars. The change in electrical resistivity at 77 K, $\delta {\rho }_{77},$ is normally used for the discussion ${\rho }_{77}$.since the signal noise ratio (S/N) at 77 K is lower than that at 293 K.

### SAXS and USAXS measurements

SAXS and USAXS were performed at a synchrotron radiation facility, BL-19B2 of super photon ring 8 GeV (SPring-8), Japan. The energy of incident X-ray beam was set to 25 keV, and the beam was irradiated with the specimen with the thickness about 0.3 mm. The scattered beam was detected by a two-dimensional detector, PILATUS 2 M, and the transmitted direct beam was blocked by a stopper. The camera length was 3044 mm and 40,774 mm for SAXS and USAXS, respectively.

SAXS/USAXS can detect the fluctuation region in electron density, since X-ray is scattered by electrons. In other word, either the atomic number of elements contained or the density (mass per volume) of the fluctuation region affects the scattering. In general, the former is attributed to the fluctuation region related to the composition and the latter is attributed to the presence of the different phases (e.g. precipitates). Unless determining the type of the fluctuation, it is difficult to determine the precise volume fraction of the fluctuation region. However, the volume fraction can be qualitatively discussed with assuming that the type of fluctuation regions is same among the specimens. The number density of fluctuation region cannot be discussed in the present study since the precise volume fraction of the fluctuation region cannot be precisely determined. Since the estimation of the size is rough error within 50% may be involved.

### HAXPES measurements

Hard X-ray photoemission spectroscopy (HAXPES) was performed at BL-46XU of SPring-8^50^. In general, X-ray photoemission spectroscopy (XPS) uses Al K $\alpha$ and Mg K $\alpha$ lines as the excitation sources, whilst HAXPES uses high-flux X-ray from third generation synchrotron radiation facility^51^. The energy of the incident X-ray beam from an undulator was 7.94 keV with a Si (111) double crystal and a Si (444) channel cut monochromator. The slit size was 0.5 mm $\times$ 30 mm. The Scienta Omicron R4000-10 kV was used as an analyzer, with the pass energy of 200 eV and the take-off angle of 80°, and the measurements were performed at R.T. The largest difference between XPS and HAXPES is the escape distance of photoelectrons, which is about several nm and a few tens nm for XPS and HAXPES, respectively. Thus, the effect of surface, such as oxidation and C contamination, is less sensitive in the case of HAXPES compared with the conventional XPS.

## Supplementary Information

Supplementary Information.

## Data Availability

ll data generated or analysed during this study are included in this published article (and its Supplementary Information files).
