# Supplementary material for: Destruction of mesoscopic chemically modulated domains in single phase high entropy alloy via plastic deformation
Source: Sci Rep. 2022 Oct 6;12:16776. doi: 10.1038/s41598-022-20932-y (PMC9537164; doi:10.1038/s41598-022-20932-y)
Supplement: Supplementary file 1 — Supplementary Information. [file 41598_2022_20932_MOESM1_ESM.pdf]

Supplementary information

SEM/EDS and STEM/EDS

Figure 1 shows SEM/EDS 2D-mapping at r = 0 %, which does not show the clear segregation of the specific elements, such as grain boundary segregation. As shown in Fig. 2, STEM/EDS 2D-mapping at r = 0 % does not also show clear modulation of the concentration of specific elements. The selected area diffraction (SAD) with [110] incident beam shows single fcc phase and no satellite spots for LCO/SRO (the inset in Fig. 2(a)). No precipitates and segregation were observed from the images.

HAXPES

Figure 3 shows HAXPES spectra of (a) Cr 2p, (b) Mn 2p, (c) Fe 2p, (d) Co 2p, (e) Ni 2p, and (f) valence band.

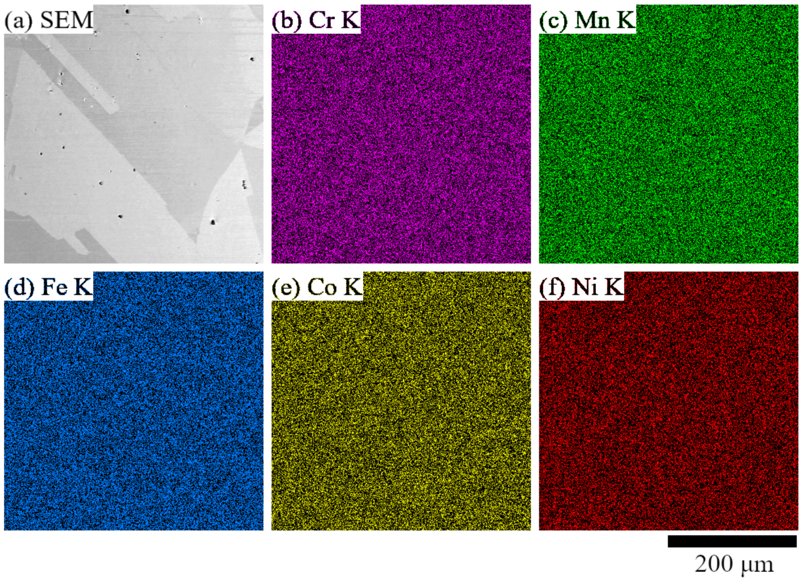

Fig. 1 SEM/EDS observations of (a) SEI and EDS map of (b) Cr K, (c) Mn K,(d) Fe K, (e) Co K and (f) Ni K.

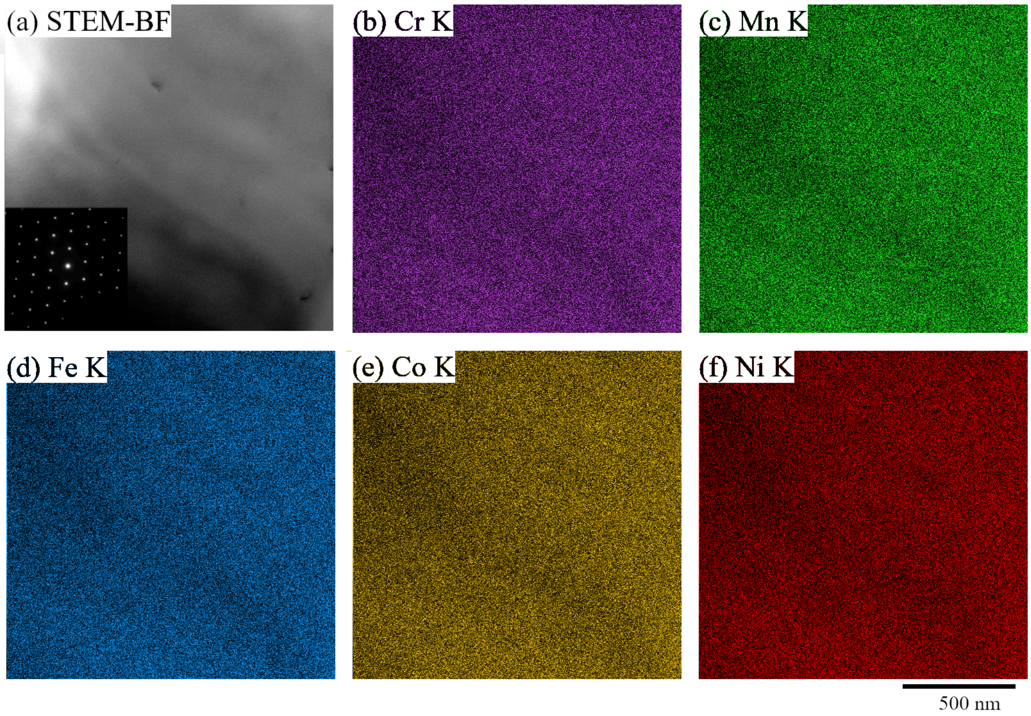

Fig. 2 STEM observation of (a) BF image and [110] incident SAD pattern (inset), and EDS map of (b) Cr K, (c) Mn K,(d) Fe K, (e) Co K and (f) Ni K.

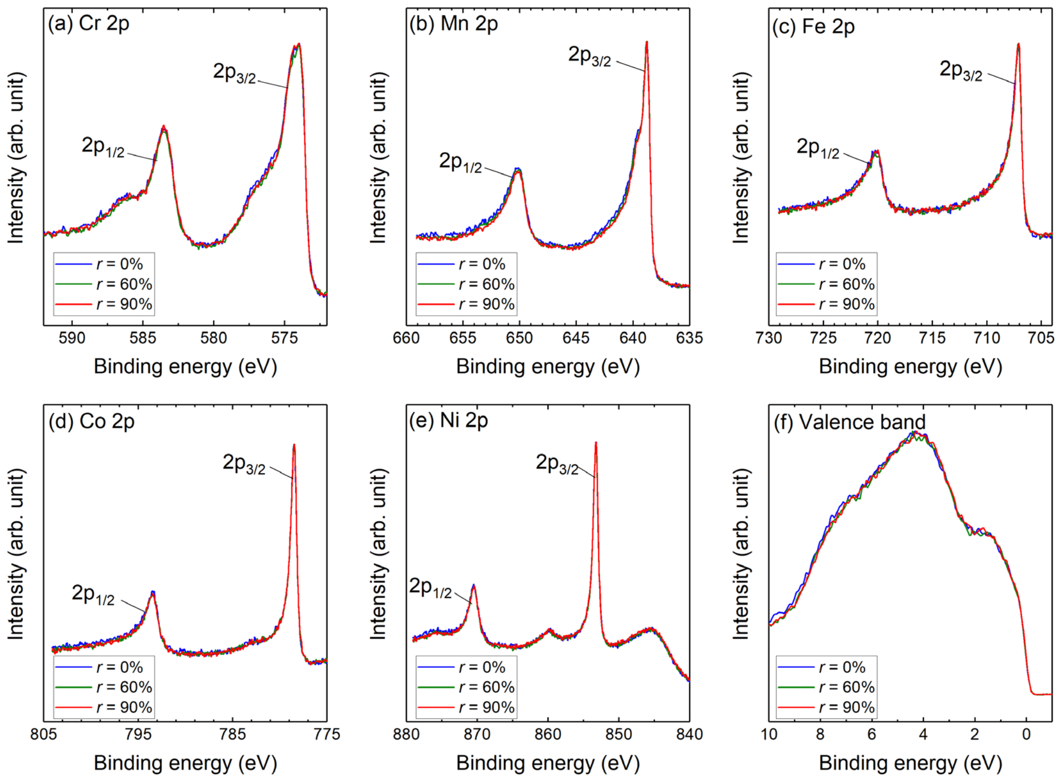

Fig. 3 HAXPES spectra of (a) Cr 2p, (b) Mn 2p, (c) Fe 2p, (d) Co 2p, (e) Ni 2p, and (f) valence band.
